# Methodologies for In Vitro Cloning of Small RNAs and Application for Plant Genome(s)

**DOI:** 10.1155/2009/915061

**Published:** 2009-06-15

**Authors:** Eric J. Devor, Lingyan Huang, Abdusattor Abdukarimov, Ibrokhim Y. Abdurakhmonov

**Affiliations:** ^1^Department of Obstetrics and Gynecology, University of Iowa Carver College of Medicine, 3234 MERF, Iowa City, IA 52242, USA; ^2^Molecular Genetics, Integrated DNA Technologies, 1710 Commercial Park, Coralville, IA 52241, USA; ^3^Center of Genomic Technologies, Institute of Genetics and Plant Experimental Biology, Academy of Sciences of Uzbekistan, Yuqori Yuz, Qibray region Tashkent district, Tashkent 111226, Uzbekistan

## Abstract

The “RNA revolution” that started at the end of the 20th century with the discovery of post-transcriptional gene silencing and its mechanism via RNA interference (RNAi) placed tiny 21-24 nucleotide long noncoding RNAs (ncRNAs) in the forefront of biology as one of the most important regulatory elements in a host of physiologic processes. The discovery of new classes of ncRNAs including endogenous small interfering RNAs, microRNAs, and PIWI-interacting RNAs is a hallmark in the understanding of RNA-dependent gene regulation. New generation high-throughput sequencing technologies further accelerated the studies of this “tiny world” and provided their global characterization and validation in many biological systems with sequenced genomes. Nevertheless, for the many “yet-unsequenced” plant genomes, the discovery of small RNA world requires in vitro cloning from purified cellular RNAs. Thus, reproducible methods for in vitro small RNA cloning are of paramount importance and will remain so into the foreseeable future. In this paper, we present a description of existing small RNA cloning methods as well as next-generation sequencing methods that have accelerated this research along with a description of the application of one in vitro cloning method in an initial small RNA survey in the “still unsequenced” allotetraploid cotton genome.

## 1. Introduction

In the 1990s two independent discoveries opened up the previously unsuspected world of noncoding RNAs (ncRNAs). The phenomenon of RNA interference (RNAi) was being uncovered as cosuppression in plants [1, 2], quelling in fungi [3, 4], and RNAi in nematodes [5] through the 1990s and at least the broad strokes of the mechanism were elucidated by the turn of the 21st Century [6]. At the same time, another curious phenomenon was being observed by Victor Ambros, Gary Ruvkun, and colleagues in nematodes [7, 8]. Like RNAi, this phenomenon, initially called short temporary RNA (stRNA), was at first regarded as a one-off curiosity but, again like RNAi, persistence paid off with the explosive validation of the microRNA (miRNA) [9–12]. The two worlds of RNAi and miRNAs merged when it was observed that both RNAi and miRNAs employed the same mechanism to carry out their mission of regulating eukaryotic gene expression [13]. 

Over the past several years RNAi has become a powerful tool for understanding the role played by dozens of plant and animal genes in a wide range of cellular processes, both normal and pathogenic [14]. Moreover, RNAi is proving to be a potentially powerful tool in attacking pathogenic cellular processes [15]. Similarly, the world of miRNAs has grown from the two original nematode “genes” to now number more than one thousand loci in plants and animals and their role in regulating cellular processes has expanded to a point where virtually all normal and pathogenic cellular processes are affected at some point by one or more of these tiny entities. Hence, the discovery of miRNAs represents a hallmark in RNA science for understanding RNA-dependent regulation of many complex biological processes such as development, function of metabolic pathways, cell fate and death [16]. 

In addition, the universe of small RNAs has expanded to include not only miRNAs but new classes including endogenous small interfering RNAs (siRNAs), 21U RNAs, and Piwi-interacting RNAs (piRNAs) [17]. Of these small RNA classes, only miRNAs form a characteristic thermodynamically stable hairpin structure. That stable hairpin makes miRNA prediction in sequenced genomes a relatively tractable exercise. On the other hand, de novo finding of miRNAs in species whose genomes have yet to be sequenced and discovering new classes of small RNAs must still rely upon in vitro cloning from purified cellular RNAs. Thus, reliable and reproducible methods for cloning small RNA species are of paramount importance and will remain so into the foreseeable future. Here, we present a compilation of extant small RNA cloning methods, options for sequencing, and some of the small RNA results that we have obtained in the “still unsequenced” allotetraploid cotton genome.

## 2. Small RNA Cloning Strategies

There are a number of strategies that have been proposed for cloning small RNAs. Before discussing these, however, there is one factor common to all of them that is essential to be aware of. Small RNAs, whether from plant cells, animal cells, or other sources, represent a small fraction of the total RNA mass present. Agilent Technologies quantifies the quality of cellular RNA in the form of their RNA Integrity Number (RIN). Very high quality intact RNA has a RIN of 10.0 and the lower the RIN, the more degraded the RNA. RIN values between 6.5 and 10.0 represent a continuum of acceptable to excellent RNAs. Using RIN as the point of departure, Agilent assessed the relative fraction of total RNA that is within the small RNA size range in forty tissues from human, mouse, and rat [18]. 

**Figure 1:**
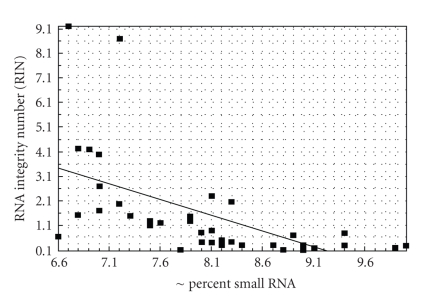
Linear regression of total RNA quality (RIN) and the relative mass of the small RNA population determined for forty human, mouse and rat tissues. A significant negative correlation coefficient, *r* = −0.58, *P* < .01, df = 38, derived from the regression indicates that total RNA quality is an essential component of small RNA cloning in that higher quality RNA retains a more pure small RNA fraction but, as a corollary, that enrichment of the small RNA fraction prior to cloning is crucial to success.

**Figure 2:**
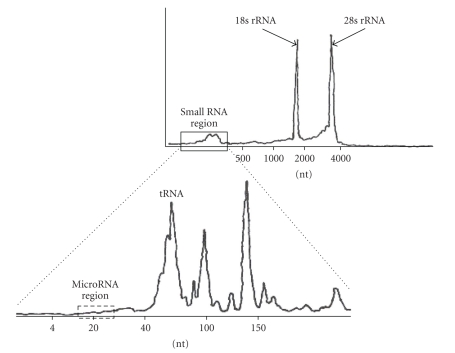
Mass profile of human RNA. Here, the absolute mass fractions of RNAs up to 4000 nt in length are shown. The position and composition of the small RNA region, defined as that portion of the total RNA mass that is between 0 and 200 nt long are highlighted. It can be seen that, even within the small RNA region, the microRNA region lying between 18 and 26 nt, is a very small fraction of the total. Figure adapted, with permission, from Agilent Technologies.

The results, summarized in Figure 1, show two important features. First, for all but five tissues, the relative mass of small RNAs is below 3% and, second, there is a significant negative correlation (*r* = −0.58; *P* < .01, df = 38) between overall RNA quality as assessed by RIN value and relative small RNA mass. Clearly, increasing amounts of RNA degradation will introduce a greater mass of small fragments that lie in the true small RNA zone. This will result in a greater mass of competing RNA that will make it more and more difficult to see the real small RNAs that are the targets of interest even if the majority of the degraded RNAs are themselves unclonable by some of the methods discussed below. While there will be variation from RNA source to RNA source, it is clear that larger RNA components like mRNAs, rRNAs, and tRNAs, comprise by far the bulk of the total RNA and that the relative mass of the true small RNA fraction should and will be the smallest in very high quality RNA. A generalized RNA mass profile for high RIN RNA is presented in Figure 2. As can be seen, the true miRNA region is indeed a very small part of the total mass. Given this, it is essential to the small RNA cloning process that RNA quality, as assessed by measures like RIN, be as high as possible and that as much of the competing RNA mass as possible be removed so that a “target-rich” small RNA component can be purified prior to starting the cloning process. 

Small RNA enrichment can be accomplished in a number of ways. One of the simplest ways is to simply run a sample of total RNA on a denaturing polyacrylamide gel (dPAGE) and excise the area of the gel containing the small RNA fraction (see the appendix). The problem with this method is that the enriched small RNAs must be removed from the gel and purified for further manipulations and this routinely results in a substantial loss of what is already a small amount of mass to begin with. There are ways to minimize this loss of material and we will discuss one of these in the next section. Other methods for enriching the small RNA fraction have been developed including column capture and release methods like the mirVana protocol from Ambion and the timed size exclusion method, represented by the flashPAGE fractionator system, also from Ambion. The point is that, whatever method is employed, the small RNA fraction of total cellular RNA must be enriched to increase the likelihood of successfully cloning small RNAs.

**Figure 3:**
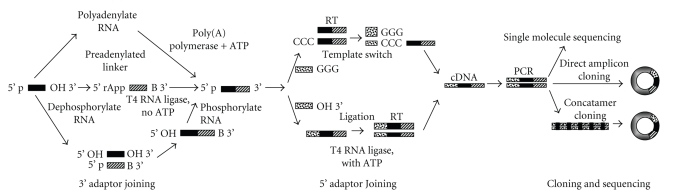
Diagram of extant small RNA cloning strategies. Following small RNA enrichment, all strategies share the same outline of first placing an adaptor on the 3′ end of the target RNAs, then placing a second adaptor on the 5′ end of the RNAs, followed by reverse transcription, amplification and cloning. Recent advances in next generation high throughput single molecule sequencing platforms have eliminated the actual cloning step but still relie to a greater or lesser extent on the same upstream methods. Figure adapted, with permission, from Berezikov et al. [19].

Once the small RNA fraction is enriched and purified, there are several ways to proceed to clone the individual small RNAs contained in the fraction. Berezikov et al. [19] reviewed the basic small RNA cloning methods. In all cases the target species for direct cloning is an RNA varying in size between 18 and 25 nucleotides (nt) having a free 3′ hydroxyl group and a free 5′ phosphate group. Although some variation exists [20], the universal initial step in the cloning process is first to ligate a 3′ adaptor sequence through the free 3′ hydroxyl. The 3′ adaptor will serve as the site for later annealing of an oligonucleotide primer for reverse transcription. As seen in Figure 3, there are several possible ways to accomplish this adaptor joining. In one option, the small RNA species are polyadenylated creating a 3′ extension [21]. However, as many small RNA species in plants have been shown to contain 2′-O-methyl modifications on their 3′ ends, this method may be of only limited utility since such modifications block polyA polymerase extension [22]. Both of the other 3′ adaptor joining options are designed to prevent later circularization of the linkered RNAs. In one variation, the RNAs are dephosphorylated prior to adaptor ligation and then rephosphorylated for subsequent processing [23, 24]. In the other variation, the 5′ end of the adaptor is preadenylated and the 3′ end blocked by a nonstandard group such as a dideoxynucleotide [10, 25]. Preadenylation of the adaptor obviates the need to dephosphorylate the target RNAs because the adaptor joining via T4 RNA Ligase can be carried out in the absence of ATP. Given the obvious advantage that this method confers by reducing the number of operations required to process target RNAs, New England BioLabs (NEB) has introduced a truncated T4 RNA Ligase that specifically reacts with preadenylated 3′ linkers [25–27]. Regardless of the method chosen, however, producing a stable and reactive 3′ linkered small RNA population is the goal of the first step in cloning. 

The next phase of cloning is to join a second adaptor to the small RNA population. This time, the adaptor is joined to the 5′ end. As shown in Figure 3, there are now but two ways to do this and the choice is dictated by the methods chosen for 3′ adaptor joining. If the method chosen is the polyadenylation route, then the 5′ adaptor joining method is to carry out a template switch. This method relies on the property of a number of reverse transcriptases to add a small number of nontemplated nucleotides to the 3′ ends of cDNAs. Since the nontemplated nucleotides tend to be mostly deoxycytidines, an adaptor containing a poly-G 3′ run can be used to switch the template from the miRNA to the adaptor [19]. The other path is to use a 5′ adaptor with a 3′ hydroxyl group that will ligate to the 5′ phosphate of the target RNAs. This is carried out with a T4 RNA Ligase in the presence of ATP and is followed by a reverse transcription using a primer complementary to the 3′ linker. In both cases, the resulting cDNA population is PCR amplified in preparation for cloning and/or sequencing.

PCR amplicons can be directly cloned using any one of several PCR cloning vectors or the amplicons can be processed to form concatamers which are then cloned. Concatamer formation from amplicons is a direct descendant of the Serial Analysis of Gene Expression (SAGE) methodology developed in the 1990s by Velculscu and colleagues [24, 28]. The obvious advantage of concatamer cloning is that individual clones will contain more small RNAs than the ones that will be present if the PCR amplicons are simply shot-gun cloned. This is a consideration for conventional Sanger dye-terminator sequencing but, as will be discussed later, new generation deep sequencing methods have circumvented the need for concatamers and, indeed, for cloning at all.

One aspect of the cloning methods shown in Figure 3  is that small RNAs will all contain a 5′ phosphate group following 3′ adaptor joining. This constant feature that allows for subsequent 5′ adaptor joining was believed to represent the universal state of small RNAs in vivo. In 2007, Pak and Fire [29] announced that this is not the case. Attempts to clone a specific small RNA in *C. elegans* called Cel-1 repeatedly failed even though there was ample evidence that it existed. Their persistence in uncovering the reason for Cel-1 being refractory to conventional small RNA cloning methods paid off in their discovery that Cel-1, and, now, other small interfering RNAs, was tri-phosphorylated on its 5′ end [29]. They developed an alternative method for cloning troublesome RNAs featuring the use of *two* 3′ ligations with the reverse transcription step in between the two ligations. This alternative method, named by them 5′ Ligation Independent Cloning, is completely indifferent to the state of the 5′ end of the target RNAs. The reverse transcription step following the initial 3′ adaptor ligation makes the initial 5′ end the new 3′ end with a hydroxyl group ready for a second 3′ ligation step regardless of what may or may not have been present on that initial 5′ end. The 5′ Ligation Independent Cloning option revealed that a secondary pool of small RNAs was being produced in *C. elegans* via a completely different pathway from conventional miRNAs [29].

## 3. Cloning with Adenylated Linkers

**Figure 4:**
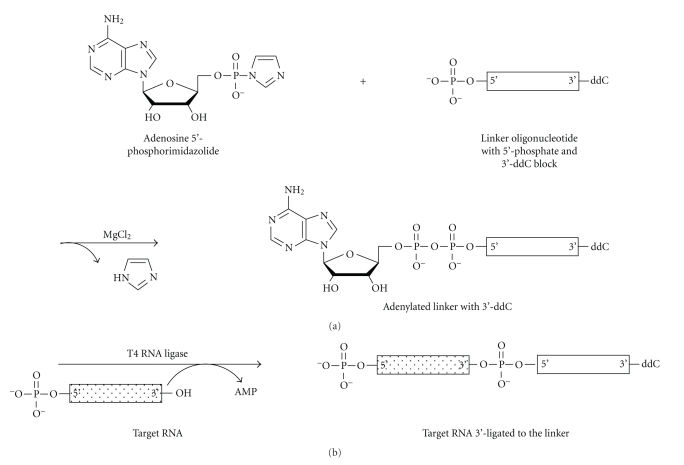
Synthesis and ligation of high efficiency 3′ adenylated cloning linkers. (a) An adenosine 5′-phosphorimidazolide is attached, in the presence of magnesium chloride, to a synthetic deoxyribo-oligonucleotide bearing a dideoxycytidine (ddC) block on its 3′ end and a free, reactive phosphate group on its 5′ end. (b) The synthetic, preactivated 3′ linker is ligated to target small RNAs in the presence of T4 RNA Ligase. This reaction is carried out with high efficiency in the absence of ATP to prevent circularization of the target RNA species prior to ligation. Reaction energy is provided by the phosphorimidazolide at the 5′ end of the linker.

While each small RNA cloning strategy has its own strengths and weaknesses, the method employing a preactivated, adenylated 3′ linker sequence, pioneered by David Bartel [10], has proved to be a readily accessible and flexible method. The adenylation of the 5′ end of a DNA oligonucleotide provides a preactivated linker that will specifically ligate to the 3′ hydroxyl group of RNA in the presence of the enzyme T4 RNA Ligase. This reaction proceeds in the absence of ATP, which is known to promote circularization of the target RNAs in solution. The 3′ end of the preactivated linker is blocked with a nonstandard base, such as dideoxycytidine (ddC), to prevent circularization of the linker. The synthesis and ligation reactions are shown in Figure 4. The synthesis reaction begins with an deoxyoligonucleotide synthesized with a 3′ block, such as ddC, and a 5′ phosphate. Adenylation at the 5′-end of the oligonucleotide is achieved through the introduction of adenosine 5′-phosphorimidazolide in the presence of magnesium chloride as the catalyst. 

Once purified, the linker, with the form rApp-(dNTP)n-ddC, will react with the free 3′ hydroxyl of an RNA in the presence of T4 RNA Ligase and the absence of ATP to create a 3′-linkered RNA plus AMP. This reaction is quite efficient so long as a relatively small mass of T4 RNA Ligase is used. Aravin and Tuschl [26] showed that the enzyme itself in commercial preparations of T4 RNA Ligase is adenylated and that this can cause circularization of the target RNA species and other unwanted side reactions that severely reduce production of the desired ligation product. A truncated T4 RNA Ligase called T4 RNL-2 truncated, that specifically and efficiently ligates adenylated linkers to RNAs in the absence of ATP without producing side reactions is available from New England BioLabs [25–27]. A number of preadenylated 3′ linkers are now commercially available. New England BioLabs offers one with a 3′ amino block and Integrated DNA Technologies (IDT) offers three linkers, each with a 3′ ddC block.

Once the target small RNAs are 3′ ligated, any unligated linkers are removed by a denaturing polyacrylamide gel electrophoresis (dPAGE) purification of the ligated material. As with initial small RNA enrichment, gel purification of the ligated RNAs is subject to substantial loss of material. One way to significantly reduce this loss is to process the acrylamide gel slice containing the RNAs using a column originally developed by Edge Biosystems for cleaning up Sanger dye terminator cycle sequencing reactions. Called Performa Columns, these spin columns will retain the acrylamide gel, salts, and urea while passing as much as 95% of the RNA into the collection tube (see the appendix). The 3′-linkered RNAs so recovered will have a 3′ end block courtesy of the linker but will retain their 5′ phosphate groups. This provides a coupling group for ligation of an oligonucleotide composed of a few 5′ DNA bases and a run of 3′ RNA bases that will ligate to the target RNAs in the presence of T4 RNA Ligase and ATP. Again, a commercial 5′ linker, called 5′ MRS, is available from IDT that is compatible with each of their 3′ linkers as well as the NEB 3′ linker.

Doubly-ligated RNAs are converted into an all DNA substrate by reverse transcription using an RT primer complementary to the 3′ linker. These cDNAs are then amplified in a PCR reaction that uses the RT primer as the reverse PCR primer and a forward PCR primer compatible with the 5′ linker. Thus, all target RNAs can be amplified for subsequent cloning using a universal PCR primer pair. Following PCR amplification the target-containing amplicons can be cloned with any one the vector systems designed for PCR cloning.

## 4. Sequencing Strategies

The generally accepted criteria for adding a new miRNA to the ever growing catalog being ably curated in miRBase [30, 31] are that the sequence of the mature 21 to 23 nt candidate is not already present among extant miRNAs, that the sequence is expressed, and that there is flanking sequence ranging in size from 60 to more than 100 nt that, with the mature sequence inside, forms a thermodynamically stable hairpin secondary structure [19, 32]. Direct cloning and sequencing from an enriched pool of small RNAs satisfies the first two of these three criteria at the same time. For this reason, sequencing is obviously a crucial part of miRNA cloning and, given that there are usually hundreds of small RNAs being expressed at various levels in tissues of interest, the more efficiently that clones can be sequenced, the better the chances of discovering new candidates. In the world of Sanger-type, dye terminator sequencing a solution is available. This solution makes use of the simultaneous sequencing capabilities of multi-capillary platforms like the GE Healthcare MEGABACE or the ABI 3730xl 96-capillary machines. On these platforms small RNAs can be sequenced either as single insert shot-gun clones (e.g., [33]) or as concatamers as shown in Figure 3. This is clearly an improvement over any previously available method but one of the most important technological advances of the post-genome era is the development of several Massively Parallel Signatures Sequencing (MPSS) [34] systems that not only produce several orders of magnitude with more quality sequences per run but also allow researchers to skip the actual cloning steps in Figure 3  altogether.

The first of the massively parallel sequencing systems to arrive on the scene was the Roche pyrosequencing platform originally developed at 454 Life Sciences [35]. This platform utilizes the phenomenon of pyrophosphate release that accompanies nucleotide incorporation to initiate a light detection reporting system based on the cleavage of oxyluciferin by luciferase [36]. The nucleic acids to be sequenced are sequestered in micron-sized emulsion PCR “reactors” following ligation of 5′ and 3′ adaptors that serve as the universal templates for clonal amplification inside the reactors. Universal adaptor ligation and subsequent clonal amplification provide an ideal opportunity to feed 5′ and 3′ ligated small RNAs directly into the sequencing flow by making “fusion primers” that incorporate both the RNA linker and Roche (454) adaptor sequences. These fusion primers would be 40-mers composed of the Roche (454) 5′ adaptor plus the 5′ linker sequences on one end and the 3′ linker plus the Roche (454) 3′ adaptor sequences on the other end (Table 1). These primers would then be used to amplify directly from the reverse transcript cDNAs. In addition, these primers can be “barcoded” so that mixed RNA populations could be simultaneously sequenced and the sequences deconvoluted later based upon the barcodes (Table 1). Similar models have already been successfully used [37, 38]. The performance obtained by the Roche 454 Life Science commercial system Genome Sequencer (GS-FLX) platform of 99.5% accuracy and average read lengths of over 250 bp resulting in outputs exceeding 200 000 reads with acceptable Phred values (a DNA sequence quality score) is ideal for searching genomes for new small RNAs and, indeed, such studies have already resulted in the discovery of the curious 21U RNA class of small RNA in *C. elegans* [39]. According to the latest updates, current 454 FLX platform is capable of sequencing 400–600 million high-quality bases in ten hours with an average of ~400 bp long reads and a raw base accuracy of 99% (http://www.454.com/products-solutions/system-features.asp; [40]). This makes the 454 FLX platform with several hundred times higher throughput compared to the current state-of-art Sanger-based capillary sequencing system. However, current limitations of this platform compared to Sanger system are relatively shorter read length as well as challenges with sequencing of homopolymer regions. The latter limitation is due to nonterminating chemistry during pyrosequencing that introduces nucleotide substitution errors [41].

Another of the next generation sequencing platforms, based on a four-color DNA sequencing-by-synthesis (SBS), introduced by Illumina/Solexa (http://www.solexa.com/), also incorporates the use of oligonucleotide adaptor ligations to produce millions of short, ligated nucleic acid fragments that are then covalently bound to a solid surface and ultimately interrogated by reversible fluorescent terminator synthesis reactions [36, 41, 42]. In comparison with the current 454 FLX platform, Illumina/Solexa platform has a higher throughput sequencing capability that equals to 1–1.5 billions of 35 bp reads per run [41]. The read length is well suited to the 21 to 31 nt size range of the so-far known small RNA classes. Although 454 FLX and Illumina/Solexa platforms utilize the same SSB sequencing principle, the sequencing chemistries (pyrosequencing versus fluorescent-based solid phase) and consequently the limitations of two systems are substantially different [41]. The major limitation of the Illumina/Solexa platform with regard to small RNA applications is also the potential for nucleotide substitution errors though the use of fluorescent-based solid phase dye terminators makes homopolymeric runs less problematic [41]. 

Also in the small RNA size range of read lengths is the Applied Biosystems' Sequencing by Oligo Ligation and Detection (SOLiD) platform. SOLiD is the combination of MSSP and polymerase colony (polony) sequencing [41, 42, 44, 45] that creates emulsion PCR generated clonal amplicons on 1 *μ*m magnetic bead from genomic fragments. Sequencing-by-ligation is carried out on enriched beads through the repeated cycles of ligation of mixture of sequencing and 8-mer fluorescently labeled oligonucleotide probes to the amplicons and detecting the color [36, 42, 45]. The SOLiD system delivers 1–3 billion bases read per run or 200–300 million bp sequence data per day with 25 to 35 bp lengths and a raw base accuracy of 99% [41, 42]. This comparatively higher throughput level of SOLiD system is achieved by using smaller beads and random array format compared to 454FLX system (26 *μ*m and ordered format). However, similar to the Illumina/Solexa system, there is a potential for incorporating substitution errors and with the shorter read lengths these can be misleading when sequencing small RNAs [41]. 

Although yet-unavailable for many small scale molecular biology laboratories with limited funding constraints, these new generation sequencing platforms are already being widely used by plant researchers to characterize plant small RNAs. A pioneer MPSS effort has revealed more than 2 million small RNAs from flower and seedling tissues of model plant *Arabidopsis thaliana*, yielding over 75 thousand distinct sequence signatures [46]. The small RNAs in various Arabidopsis [47, 48] and maize [49] mutant backgrounds were deep sequenced and characterized. Recently, small RNA/miRNA pools in rice were characterized using these next generation sequencing platforms [50, 51]. Chellappan and Jin [52] published an excellent review of small RNA cloning and discovery methodology in plants and have compared the deep parallel sequencing of small RNA libraries using aforementioned 454, Illumina/Solexa, and SOLiD technologies. 

In general, all of the next generation sequencing technologies offer unprecedented sequencing depth in a very short time. The power of these platforms is that they are only capable of finding all or nearly all of the small RNAs expressed in a particular tissue but they can do so in a quasiquantitative manner due to the enormous number of sequence reads generated, dramatically reducing the cost. However, since next generation sequencing platforms are still under development and most likely will be improved for higher throughput and accuracy at reduced cost, at present, the suitability of any particular platform for small RNA sequencing comes down to study objectives and the availability of the platforms.

## 5. Application: Cotton Small RNAs

There are many excellent methods available that utilize known microRNA sequences for the purpose of determining both absolute and relative expression levels in various tissues and under various conditions. These methods primarily focus upon either quantitative, or real-time, PCR or microarray hybridizations. However, as noted above, the primary objective of small RNA cloning is different, it is discovery of both new miRNAs and new classes of small RNA. In this final section, we will briefly present results that we have obtained using an adenylated cloning linker strategy (refer to [33, 53] for detailed protocol) to investigate the pool of small RNA signatures and discover plant small RNAs in root tip and developing ovule tissues of a widely grown Upland cotton *G. hirsutum* L. These results are initial surveys, but the first effort of “wet-bench” works toward studying the small RNA world for a complex “still unsequenced” allotetraploid cotton genome. 

The genus *Gossypium* L. includes approximately 45 diploid A-G to K genomic groups [54] and 5 allotetraploid (AD_1_–AD_5_ lineages formed by A- and D-genome hybridization about 1-2 million years ago) species [55]. The genomes of allotetraploid cottons have a chromosome complement of 2*n* = 4X = 52, a haploid genome size of 2200–3000 Mb DNA, and a total recombination length of approximately 5200 cM (an average of 400 kb per cM) [56]. Accordingly, allopolyploid cotton genomes are one of the largest plant genomes with its complex nature, and are an important model system to study fundamental biological studies in plants [57]. Furthermore, cotton fiber is regarded as a unique single-celled model system to study cell growth initiation, elongation, differentiation and cellulose biosynthesis in plants [57–59]. 

As of February 2009, a search of the GenBank nucleotide database for *Gossypium* revealed a total of 452, 634 nucleotide sequences, corresponding to an 8, 239 core subset of nucleotide, 375, 447 Expressed Sequence Tag (EST), and 68948 Genome Survey sequence (GSS) records (http://www.ncbi.nlm.nih.gov; searched on February 16, 2009). Efforts toward sequencing entire cotton genome(s) are in progress [55] and the smallest genome, *G. raimondii * (D_5_), will soon be completely sequenced and available for researchers [60]. Nevertheless, one of the major present sources of cotton genomic sequences, available through GenBank, only corresponds to an 11.4 Mb of cotton genome [57]. This is a serious obstacle for systematically searching the cotton genome for small RNA/microRNA signatures although several investigators have reported initial efforts to identify these tiny elements in cotton using *in silico* bioinformatics analysis [61–63]. This underlies the necessity for wet laboratory cloning of cotton small RNA sequences for de novo discovery of unique small RNAs and microRNAs from various tissues in cotton, which then subsequently will be validated with availability of a complete DNA sequence of cotton genome(s) [33]. 

**Figure 5:**
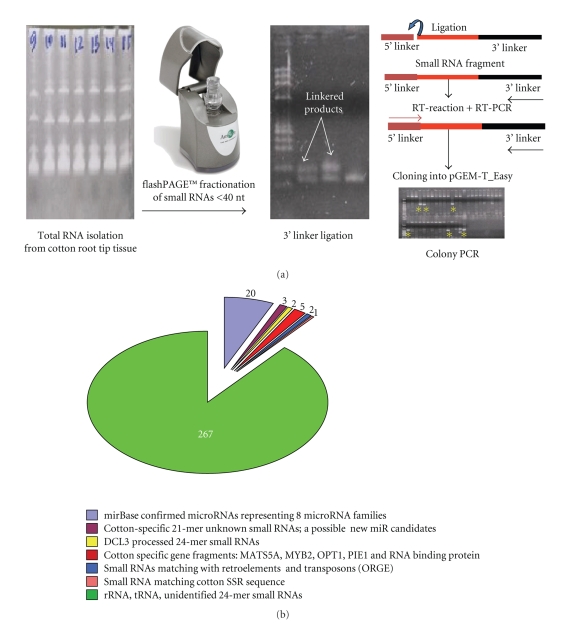
Size-directed cloning of small RNAs from cotton root tips: (a) cloning procedure stages from a total RNA isolation, small RNA fractionation, 3′ and 5′ linker ligation, and sequencing; (b) annotation of cotton root tip small RNA pools where specific group of small RNAs is color-coded for simplicity.

Using the adenylated cloning linker strategy outlined above, we have conducted an initial survey of small RNA content in the 3–5 days old root tip tissue of Texas-Marker-1 (*G. hirsutum* standard line) and sequenced ~ 300 individual colonies with the 3′ and 5′ specific linker ligated small RNA inserts [64]. Our sequencing efforts have confirmed 20 microRNA signatures from 8 families including miR-156 (7), miR-156* (1), miR-166 (4), miR-167 (1), miR-168 (1), miR-169 (2), miR-171 (2), miR-396 (1), and miR-457 (1), suggesting their involvement during early root development of cotton seed germination process (Figure 5). These very abundant micro-RNAs have known targets including transcription factor and stress response genes in other plants, and miR-156 and miR-166 are considered two of the largest and oldest miRNA families in plants [65]. In addition, we found several unidentified 21-mer small RNAs that possibly have a potential to be cotton-specific microRNAs. We also have several 24-mers that match DCL3 processed small RNAs in Arabidopsis and many unidentified 24-mers that might also be DCL3 processed small RNAs in cotton. Moreover, we found several gene-specific fragments. Two (+/−) gene hits that are notable are the Ashbya gossypii OPT1 gene and a hit on MYB2. Thus, the results of our initial attempts using size-directed small RNA cloning strategy demonstrated that the cloning method does work for finding small RNAs/microRNAs in cotton. They also confirmed the difficulty of finding plant microRNAs since we only have 20 microRNAs, representing only 8 loci, in more than 300 sequenced clones from cotton root tissue small RNA library.

**Figure 6:**
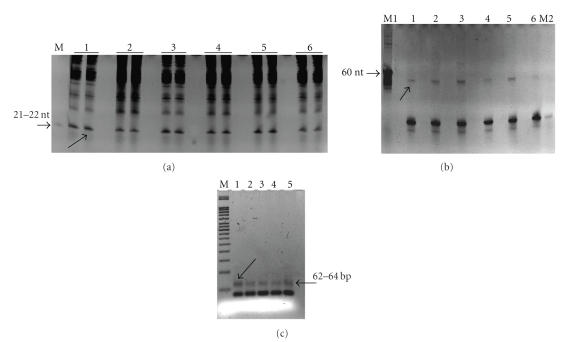
Isolation and cloning of small RNAs from cotton ovule tissue libraries [33]: (a) the example of 15% denaturing PAGE electrophoresis of total RNA from developing ovules at different DPA (0 to 6), spiked with 10 pmoles of the miSPIKE (Integrated DNA Technologies) 21-mer control RNA, M-21 nt RNA size control; (b) the example 15% denaturing PAGE electrophoresis of 3′ end linkering reaction for small RNAs from developing ovules at different DPA (0 to 6), M1–62 nt RNA size control, M2–21 nt small RNA size control; (c) 2% high-resolution agarose gel picture where RT-PCR product of 3′ and 5′ end linker ligated small RNAs of ovules was loaded, M-50 bp size ladder. Arrows indicate the small RNA fraction in (a), and linker ligated small RNA products ((b) and (c)).

**Figure 7:**
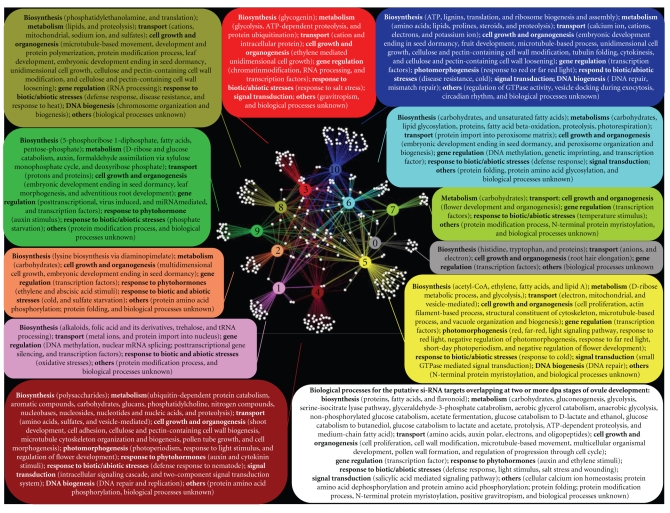
Annotation of biological processes targeted by abundant copy (>5 copies) candidate siRNAs of developing ovules in cotton. To better visualize the specific and overlapping putatively targeted proteins at 0 to 10 DPA ovules, Cytoscape [43] was used to generate genetic interaction networks of putative targets at different DPA stages of ovule, where each node (DPA) and its edges (targeted proteins) were colored. The interaction networks were depicted using Cytoscape's “spring embedded layout algorithm” for both full protein target dataset and protein groups targeted by only abundant copy candidate siRNAs, importing the “simple interaction format (SIF) files” into Cytoscape. SIF files were created based on specific and overlapping target protein information for 0–10 DPA ovule stages [33].

Recently, using the same size-directed small RNA cloning strategy with adenylated linkers, we have characterized [33] the small RNA sequence signatures in eleven postanthesis (DPA) periods of fiber development (0–10 DPA) (Figure 6). Sequencing more than 6500 individual colonies from 11 ovule small RNA libraries, we identified nearly 2500 candidate small RNAs comprising of 583 unique sequence signatures of 21–24 nt size range. As reported by Abdurakhmonov et al. [33], results showed (1) the presence of only a few mirBase-confirmed plant microRNAs (miR172, miR390 and ath-miR853-like), and these were differentially represented in specific DPA periods of ovule development. (2) The vast majority of sequence signatures were expressed in only specific DPA period and this included nearly all of the 24 nt sequences, Further, they showed (3) the existence of specific pattern of sequence diversity and abundance between 0–2 to 3–10 DPA periods, possibly corresponding to the transition of fiber initiation to elongation phase of fiber development. Further, target predictions *in silico* using ovule-derived small RNA sequences putatively indicated their involvement in numerous important biological processes including processes involving previously reported fiber-associated proteins (Figure 7). Results collectively demonstrate that the initiation and elongation stages of cotton fiber development are at least partially regulated by specific sets of small/microRNAs [33]. However, to get a better picture of cellular mechanisms of small RNA network during fiber development process, there is urgent need for so-called “deep sequencing” efforts of small RNA pools using next generation sequencing platforms [36, 49] that will undoubtedly increase multi-DPA representation of small RNAs.

## 6. Conclusions

The discovery of the world of small, regulatory RNAs has provided geneticists with a phenomenal array of opportunities as well as questions. This discovery has also led to the development of a powerful set of new molecular tools that can be used to answer those questions and take full advantage of those opportunities. The techniques built around RNA interference, real-time PCR, and microarrays allow an unprecedented level of precision in unraveling the mechanisms of gene expression and regulation. So, too, have the developments in small RNA cloning and next generation DNA sequencing discussed here opened previously barred windows on genome organization that will continue to feed into the functional genomics pipeline. The size-directed small RNA cloning strategy using adenylated linkers, highlighted with its application for the “yet-unsequenced” cotton genome small RNA characterization, is an efficient methodology for studying these tiny molecules in various plant genomes, especially suitable for the “small-scale” plant genome laboratories worldwide, that lack access to the still-expensive next generation sequencing platforms. 

## Figures and Tables

**Figure 8:**
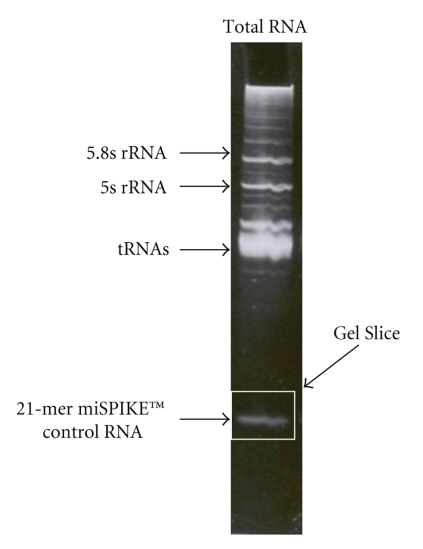


**Table 1:** Examples of Roche (454) fusion primer sequences and a set of simple bar-coded Roche (454) fusion primer sequences based upon the 3′ and 5′ linkers in the IDT miRCat Small RNA Cloning Kit.

miRCat linker-specific PCR primers:	
Forward	5′-TGGAATTCTCGGGCACC-3′
Reverse	5′-GATTGATGGTGCCTACAG -3′

Roche (454) fusion primers:

Forward	5′-GCCTCCCTCGCGCCATCAGTGGAATTCTCGGGCACC-3′
adaptor A
Reverse	5′-GCCTTGCCAGCCCGCTCAGGATTGATGGTGCCTACAG-3′

adaptor B
Simple fusion primer bar-coding scheme (6 of 256 possible sequences):

5′-GCCTCCCTCGCGCCATCAGGTACTGGAATTCTCGGGCACC-3′	
5′-GCCTCCCTCGCGCCATCAGCGATTGGAATTCTCGGGCACC-3′	
5′-GCCTCCCTCGCGCCATCAGTCGATGGAATTCTCGGGCACC-3′	
5′-GCCTCCCTCGCGCCATCAGATGCTGGAATTCTCGGGCACC-3′	
5′-GCCTCCCTCGCGCCATCAGCCTCTGGAATTCTCGGGCACC-3′	
5′-GCCTCCCTCGCGCCATCAGTGGATGGAATTCTCGGGCACC-3′	

## Appendix

### A. RNA Recovery from Denaturing PAGE Using DTR Columns

1. Run total RNA spiked with 10 pmoles of the miSPIKE (Integrated DNA Technologies) 21-mer control RNA on a 12% to 15% denaturing PAGE (7 M Urea) for 90 minutes at 275 V (be sure to monitor the gel so that the small fragments do not run off).
2. Stain the gel with GelStar nucleic acid stain (Lonza Cat. No. 50535) and place on uV light box.
3. Select RNA fragment(s) to be purified and cut it (them) from the gel as shown in Figure 8.
4. Place the gel slice in a 1.5 mL tube and crush with a glass rod. (Note: we have had very good results using the 1.5 mL tubes and disposable pestles from Kontes Glass Company).
5. Add 200 *μ*L IDT sterile, nuclease-free water and continue to crush the gel into a fine slurry. Place the tube at 70°C for 10 minutes.
6. Following manufacturer's recommendations, prepare a Performa DTR column for each gel slice.
7. Vortex the gel slurry, transfer the entire volume onto the column and spin at 3000 rpm for 3 minutes.
8. Discard the DTR column.
9. Add 3 *μ*L 10 mg/ml glycogen, 25 *μ*L of 3M NaOAc (pH5.2), and 900 *μ*L ice cold 100% EtOH to the eluent. Mix by inversion and place at −80°C for 20 minutes.
10. Spin tubes at full speed (≥10 000 rpm) for 10 minutes to pellet the RNA. Pour off the supernatant and dry the pellet.
11. Proceed to next procedure/application (e.g., miRCat protocol).

This protocol successfully removes the Urea and other salts with substantially less loss of RNA than is seen with conventional crush and soak methods followed by NAP-5 column desalting or by dialysis methods. Detail list of small RNA cloning products and protocol for miRCat can be found from IDT product manual at (http://www.idtdna.com/Support/Technical/TechnicalBulletinPDF/miRCat_User_Guide.pdf).
